# Mucinous tubular and spindle cell carcinoma of the kidney: a report of seven cases

**DOI:** 10.1186/s12885-023-11252-z

**Published:** 2023-08-30

**Authors:** Chunxiang Ling, Ru Tan, Jiamei Li, Jizhen Feng

**Affiliations:** 1 Department of Radiology, Shandong Provincial Hospital Affiliated to Shandong First Medical University, Jingwu Road 324, Jinan, 250021 Shandong Provincial China; 2 Department of Pathology, Shandong Provincial Hospital Affiliated to Shandong First Medical University, Jingwu Road 324, Jinan, 250021 Shandong Provincial China

**Keywords:** Mucinous tubular and spindle cell carcinoma, Renal cell carcinoma, Imaging features, Computed tomography, Prognosis

## Abstract

**Objective:**

To further analyse the imaging features and tumour outcomes of mucinous tubular and spindle cell carcinoma (MTSCC) of the kidney.

**Materials and methods:**

The current study retrospectively reviewed the clinical information of seven patients diagnosed with MTSCC at our institution from January 2011 to March 2023.

**Results:**

The median age at diagnosis was 52 years (range, 32–66 years) and the majority of patients were female (71.4%). On conventional abdominal ultrasound, the majority of the tumours (5/7) were heterogeneous hypoechoic or slightly hypoechoic. Colour Doppler flow imaging showed blood flow within the tumour in 2 cases and peripheral blood flow signal in 1 case. On non-enhanced CT, all tumours had a spherical or ovoid shape, with an expansile growth mode, and had clear or unclear boundaries with the surrounding renal parenchyma. The tumours were either partially exophytic (n = 4) or parenchymal (n = 3), while no cases of completely exophytic tumour was observed (n = 0). On contrast-enhanced CT, the majority of tumours (5/7) showed a heterogenous pattern of enhancement and the mean tumour diameter was 6.7 ± 4.4 cm (range, 2.1–16.8 cm). All patients underwent partial or radical nephrectomy for pT1a (42.9%), pT1b (28.5%), pT2 (14.3%) or pT3b (14.3%) stage. Among these, 1 patient (14.3%) had a level I tumour thrombus at diagnosis and died of disease 24.5 months later. The remaining patients had no recurrence or metastasis.

**Conclusion:**

MTSCC is not universally indolent, which tends to occur in female patients of a broad range of ages. MTSCC is a hypovascular renal tumour, which is different from clear cell renal cell carcinoma (RCC); however, it is difficult to distinguish MTSCC from other hypovascular RCC subtypes because of the overlap of their imaging characteristics.

## Introduction

Renal cell carcinoma (RCC) is the most mortal of all the common tumours of urological system. The classification of RCC has experienced great change over the lately 30 years. Mucinous tubular and spindle cell carcinoma (MTSCC) is a rare type of RCC, which is one of the 20 subtypes of RCC included in the 2022 World Health Organization (WHO) classification [1]. Pathologically, MTSCC is composed of a mixture of tubular and spindle cell components with variable amounts of extracellular mucin in the stroma [2, 3], which is regarded as a low-grade carcinoma. The tubules are round, ovoid, or elongated with a collapsed central lumen, which are usually firmly packed and arranged in parallel, sometimes coalescing into cord-like structures or even forming a solid growth mode. MTSCC appears to originate from the distal nephron or may be a variant of papillary RCC with proximal tubule origin [2]. MTSCC tends to occur in female patients of a broad range of ages [2]. The majority of patients with MTSCC are discovered accidentally during abdominal imaging because of other unrelated reasons, only a few tumours were discovered during workup for investigation of haematuria, flank pain or mass [2]. Because MTSCC is rare, references are limited. To the best of our knowledge, there are no unified diagnostic criteria, especially for the imaging diagnosis of MTSCC and the outcome of the MTSCC. Therefore, it is necessary to collect more clinical and imaging characteristics of MTSCC in order to improve the diagnosis and treatment of MTSCC. The present study retrospectively reviewed the clinical information of patients that were diagnosed with MTSCC at the Shandong Provincial Hospital Affiliated with Shandong First Medical University (China) from January 2011 to March 2023 with the aim of further analysing the imaging features of MTSCC as well as the tumour outcomes of the disease.

## Materials and methods

### Participants

The present study retrospectively analysed all patients diagnosed with MTSCC who were treated at the Shandong Provincial Hospital Affiliated with Shandong First Medical University (China) from January 2011 to March 2023. To confirm the diagnosis of MTSCC, all available pathological section were re-reviewed by an experienced genitourinary pathologist, as indicated in the WHO classification [1].

### Data collection

The pathology database of our institution was searched with keywords “mucinous tubule”, “spindle cell”, and “renal cell carcinoma” from January 2011 to March 2023. Seven cases of MTSCC were collected and confirmed by two genitourinary pathologists. The electronic medical record (EMR) was searched with inpatient number in order to record clinical information, surgical procedures and imaging examinations of the patients. The image information was obtained from the picture archiving and communication system ( PACS ) of the imaging centre at our institution. The enrolled patients were followed up by telephone. General clinical information, related image data, therapy received, and histological and prognostic data were collected. All patients received ultrasound and preoperative CT scans of the abdomen that were available for review. Recorded relevant ultrasound and radiologic data were re-reviewed by two radiologists and two sonographers.

Tumours were considered exophytic if ≥ 50% of the mass was deemed to be outside of the normal renal contour, while tumours were considered partially exophytic if < 50% was outside of the renal contour. Tumours were considered endophytic if completely localized within the renal parenchyma without any recognizable bulge on the contour of the kidney.

Surgical data were reviewed and the tumour stage was assigned following the Tumour, Node and Metastasis classification system established by the American Joint Committee on Cancer in 2017 [4]. Other pathological details including tumour size, margin status, necrosis, haemorrhage, sarcomatoid changes, fat invasion and invasion of adjacent organs were also recorded.

Follow-up was different based on disease stage, treatment and comorbidity. Serial telephone follow-up was obtained with the patient’s general condition, laboratory tests (blood biochemistry and urine routine) and imaging examinations (abdominal ultrasound, CT or MRI, and chest CT), and data regarding recurrent or metastasis was also recorded, including the time and location of recurrence as well as most recent disease status. The duration of follow-up was from the date of diagnosis until the last follow-up or death.

### Statistical method

Statistical analysis was performed using the SPSS26.0 statistical software (IBM Corp.). Data are expressed as the mean ± standard deviation, while count data are expressed in percentages.

## Results

### Patient characteristics and clinical findings

**Fig. 1:**
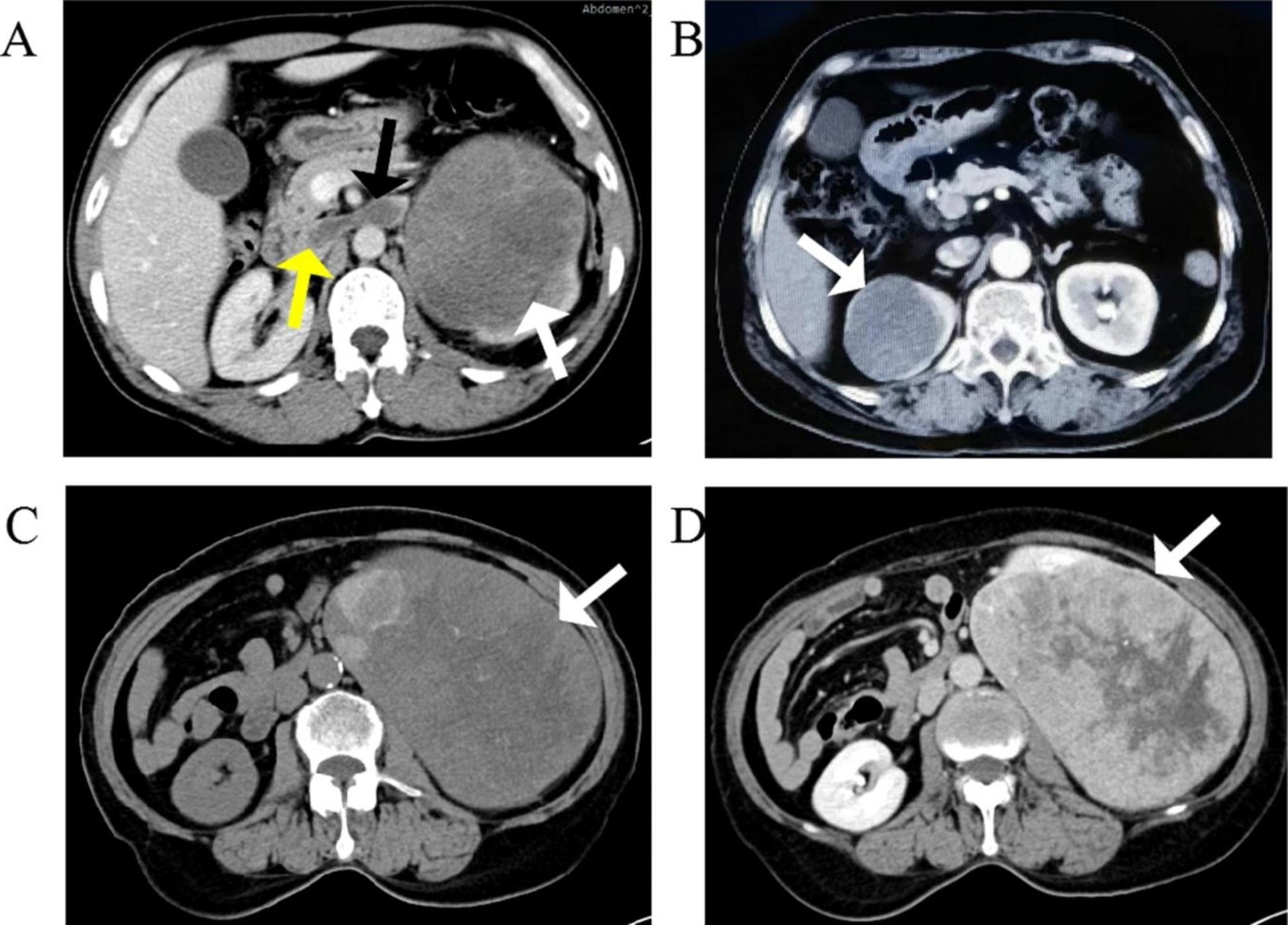
CT imaging features of mucinous tubular and spindle cell carcinoma. **(A)** Axial contrast CT-scan showed a well-demarcated left solid 12.0 cm renal lesion (white arrow) with tumour thrombus in the left renal vein (black arrow) and inferior vena cava (yellow arrow) in a 52-year-old male patient (case no. 3). **(B)** Axial contrast CT-scan showed a well-demarcated right solid 5.6 cm renal lesion (white arrow) with homogenous enhancement in a 64-year-old female patient (case no. 5). **(C)** Axial non-enhanced CT-scan showed a well-demarcated left solid 16.8 cm renal mass (white arrow) with calcification, mucous degeneration and cystic degeneration in a 66-year-old female patient (case no. 6). **(D)** Axial enhanced CT-scan showed a well-demarcated left solid 16.8 cm renal lesion (white arrow) with heterogeneous enhancement in a 66-year-old female patient (case no. 6)

Patient characteristics and clinical findings of the study group are summarized in Table 1. The median age at diagnosis was 52 years (range, 32–66 years). The majority of patients (71.4%) were female. Among seven patients, six (85.7%) were asymptomatic and incidentally diagnosed with MTSCC, while one patient (14.3%) was found to have a 12 × 12 cm renal tumour because of intermittent painless gross haematuria lasting more than 1 month and aggravating 1 week. Furthermore, tumour thrombus was found in the left renal vein and inferior vena cava at the time of diagnosis was found at the time of diagnosis (Fig. 1A). None of the patients presented with bilateral masses. The median patient follow-up time was 49.5 months (range, 1.9–132.5 months).

**Table 1:** Summary of patient characteristics and clinical findings

Parameter	N (%)
**Female**	5 (71.4%)
**Median age, years (range)**	52 (32–66)
**Symptoms**	
Incidental	6 (85.7%)
Local symptoms (e.g., hematuria, flank pain)	1 (14.3%)
Symptomatic metastases	0
Left side tumor	4 (57.1%)
Tumor size, cm, median (range)	5.3 (2.1–16.8)
Follow-up time, months, median (range)	49.5 (1.9-132.5)

### Ultrasonography features

The information derived from the ultrasound scan of all patients is shown in Table 2. Conventional abdominal ultrasound indicated that the majority of tumours seemed to be well-demarcated. The majority of the tumours (5/7) were heterogeneous hypoechoic or slightly hypoechoic. Of the two remaining, one patient showed a cysticsolid mass with scattered anechoic and punctate strong echo, while the other patient showed moderate echo. Colour Doppler flow imaging (CDFI) showed blood flow inside the tumours in 2 cases and peripheral blood flow signal in 1 case.

**Table 2:** Ultrasound characteristics of primary tumour

Patient	Echogenicity	Margin	CDFI
1	Heterogeneous slightly hypoechoic	Well-demarcated	A small amount of peripheral blood flow
2	Heterogeneous hypoechoic	Well-demarcated	No blood flow
3	Heterogeneous slightly hypoechoic	Well-demarcated	No blood flow
4	Heterogeneous moderate echo	Well-demarcated	Blood flow in the tumour
5	Heterogeneous hypoechoic	Well-demarcated	No blood flow
6	Heterogeneous cysticsolid	Well-demarcated	Dot strip blood flow in the tumour
7	Heterogeneous slightly hyperechoic	Ill-demarcated	No blood flow

### Radiologic findings

CT imaging features for all the MTSCC cases are shown in Table 3. All tumours showed an expansile growth pattern with a spherical or ovoid shape on unenhanced CT and had clear/unclear boundaries with the surrounding renal parenchyma. The growth pattern of the masses was partially exophytic (n = 4) or parenchymal (n = 3), while no case of completely exophytic tumour was observed (n = 0). The tumour size range was 2.1–16.8 cm, with a mean diameter of 6.7 ± 4.4 cm. Among the seven tumour, two were homogeneously enhanced (Fig. 1B) and their diameters were 2.1 and 5.6 cm, respectively. The majority of tumours (5/7) showed a heterogenous pattern of enhancement. Among these, two tumours showed areas of calcification, one of which displayed areas of mucous degeneration and cystic degeneration (Fig. 1C and D). No tumour showed areas of haemorrhage. Tumour embolus in the left renal vein and inferior vena cava was found only in one patient. According to the Mayo classification [5], venous tumour thrombus is defined as level 0 to level IV, the patient was classified as level I.

**Table 3:** CT imaging features for all MSTCC

Patient	Side	Size	Growth pattern			Enhancement	Calcification, necrosis or cystic components										
1	Right	2.1 cm	Parenchymal			Heterogeneous,quick and late wash-out				Yes							
2	Left	5.3 cm	Parenchymal			Heterogeneous,slow and progressively				No							
3	Left	12.0 cm	Partially exophytic			Heterogeneous,quick and late wash-out				No							
4	Left	2.1 cm	Partially exophytic			Homogenous slow and progressively				No							
5	Right	5.6 cm	Partially exophytic			Homogenous slow and progressively				No							
6	Left	16.8 cm	Partially exophytic			Heterogeneous,quick and late wash-out				Yes							
7	Right	3.0 cm	Parenchymal			Heterogeneous,slow and progressively				No							

### Treatment, pathology and outcomes

The treatment, pathology and outcomes of the patients are shown in Table 4. None of the patients underwent preoperative or neoadjuvant therapy. All patients had surgical treatment. A total of four patients (57.1%) underwent radical nephrectomy, of whom one underwent thrombectomy of the inferior vena cava and lymphadenectomy. Partial nephrectomy was performed in three patients (42.9%), and one patient without metastasis received one cycle of placebo adjuvant chemotherapy in a local hospital after operation due to excessive anxiety and impulsiveness, although it did not meet the guidelines for the management of kidney cancer.

**Table 4:** Treatment, pathology and outcomes of MTSCC

	N (%)
**Local Therapy**	
Partial nephrectomy	3 (42.9%)
Radical nephrectomy	4 (57.1%)
**pTstage**	
pT1aN0M0	3 (42.9%)
pT1bN0M0	2 (28.5%)
pT2bN0M0	1 (14.3%)
pT3bN0M0	1 (14.3%)
**Tumor Necrosis**	0
**Sarcomatoid Dedifferentation**	0

**Fig. 2:**
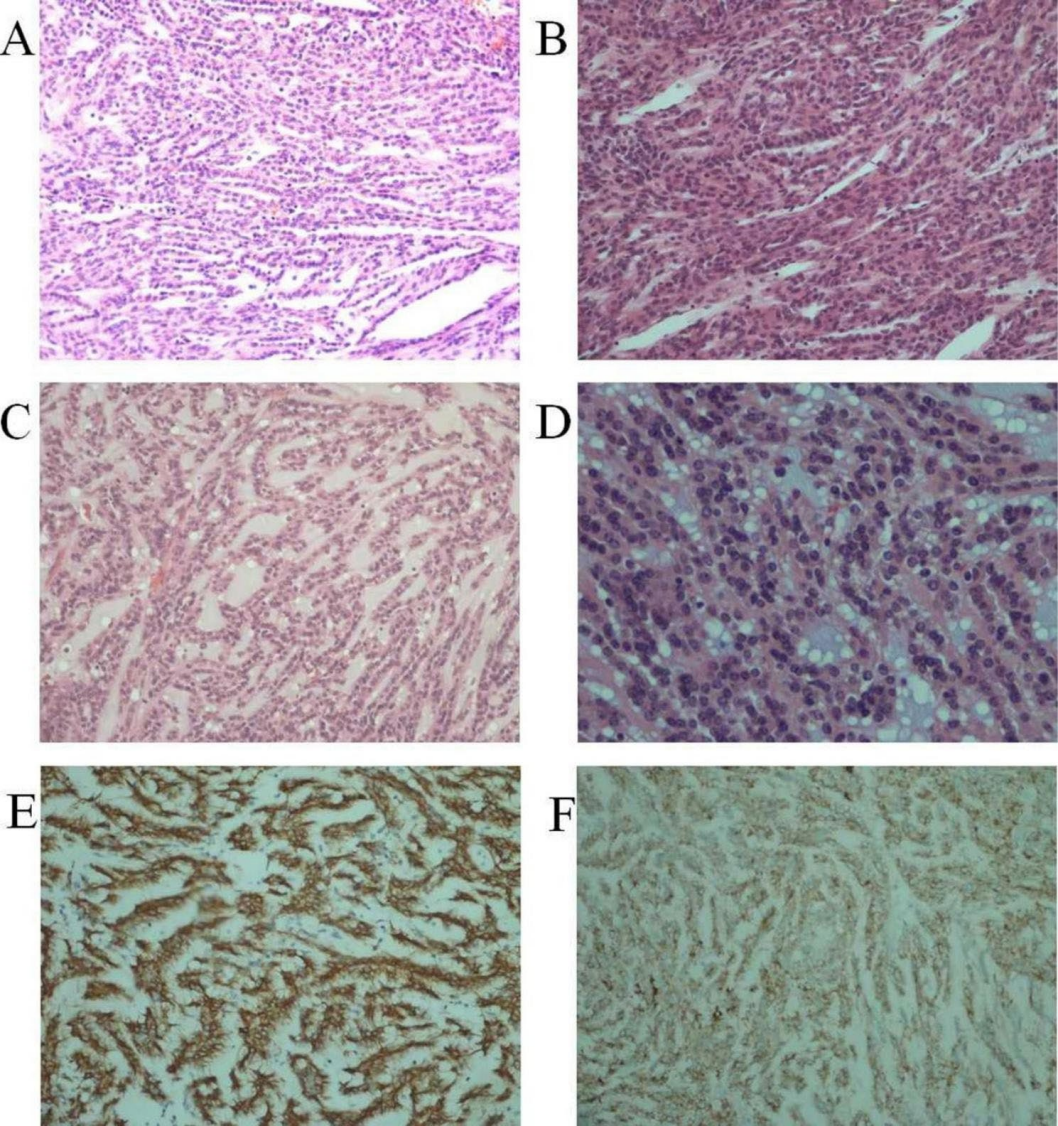
Pathological characteristics of MTSCC. **(A)** and **(B)** Tumour was composed of admixed tubular formation and spindle cells (HE; ×200 magnification). **(C)** Tumour was composed of compact tubules and spindle cells in a mucinous stroma (HE; ×200 magnification). **(D)** Tubules with low-grade cuboidal cells had eosinophilic cytoplasm, ambiguous nucleoli and rare mitosis (HE; ×400 magnification). **(E)** and **(F)** Tumour cells expressed CK7 and AMACR respectively (×100 magnification). HE, haematoxylin-eosin staining

A total of 3 cases were diagnosed with pT1aN0M0 (42.9%), 2 cases with pT1bN0M0 (28.5%), 1 case with pT2bN0M0 (14.3%) and 1 case with pT3bN0M0 with a level I tumour thrombus (14.3%). Among these, one tumour invaded the tunica fibrosa renis, and the hilar tissue and vessels. None of the cases showed lymph node metastasis and necrosis. Microscopically, all tumours consisted of tightly packed, elongated and anastomosing tubules lined by low-grade cuboidal cells merging with bland spinle cells in a myxoid stroma contaning basophilic extracelluar mucin, which were classical characteristics of MTSCC (Fig. 2A-D). Tumour cells had scant clear or eosinophilic cytoplasm, ambiguous nucleoli and rare mitosis. Ratios of the spindle cells, tubules and myxoid stroma was various in different cases. Immunohistochemically, tumours expressed CK7 (Fig. 2E), AMACR (Fig. 2F) and PAX8.

The patient with a level I tumour thrombus at diagnosis died because of the disease 24.5 months later, while the other 6 patients were still alive at the time of writing without recurrence or metastasis.

## Discussion

MTSCC is a rare subtype of RCC [1], which comprises spindle cells and tubules lined by cuboidal epithelium within varying amounts of mucinous stroma [2, 3]. According to the WHO [1], MTSCC accounts for less than 1% of all RCC. However, the exact incidence rate of MTSCC has rarely been described in previous reports, which was only explicitly presented in Xu et al. as approximately 0.52% of all RCC (22/4197) [6]. In the present study, a total of 7 cases of MTSCC accounted for 0.24% (7/2938) of all diagnosed primary RCC cases at our institution. In previous studies, MTSCC has been reported to be prevalent in female patients of a broad age range [7, 8]. In the present study, patient age ranged between 32 and 66 years (median, 52 years) and the male-to-female ratio was 2:5, which is the same as in previous studies [7, 8]. Similar to other renal tumours, MTSCC is usually asymptomatic or with incidental symptoms, such as haematuria or flank pain [9, 10]. In the current study, only one patient presented with the symptom of intermittent painless gross haematuria and was with a level I tumour thrombus, the patient eventually died because of the disease. A few patients diagnosed with MTSCC have reportedly died [7, 11–15].

Considering that MTSCC is a rare disease, to the best of our knowledge, there are only a few studies that can help define its imaging characteristics. Abdominal and/or pelvic CT or MRI with or without contrast is strongly preferred and CT triple phase is the first choice for initial diagnosis of RCC according to the guidelines of National Comprehensive Cancer Network (NCCN, 2022) and European Society for Medical Oncology (ESMO, 2022) [16, 17]. Contrast-enhanced ultrasound can be helpful in specific cases [17]. Some reports demonstrated that analysing the MRI features of MTSCC is clinically valuable [8, 18–21]. However, MRI has specific indications, which is helpful for differentiation of unclear renal masses, analysis of the extent of local advanced tumor invasion, the association with peripheral blood vessels and organs, and the presence or absence of venous cancer thrombus. All of our patients did not receive MRI scan. The present study evaluated ultrasound and CT imaging results.

In previous studies [22–24], the description of ultrasound imaging of MTSCC was uncommon. Sahni et al. [22] reported tumours that were homogenously hypoechoic with well-demarcated margins. Yan et al. [23] described the ultrasound imaging of two cases - one case with a well-demarcated, homogenous and slightly hypoechoic tumour, while the other case with a mildly heterogeneous and slightly hyperechoic tumour. Zhang et al. [24] showed the ultrasound imaging of 6 cases that were mainly hypoechoic. In a single-centre study, the majority of tumours were well-demarcated, homogenously hypoechoic and heterogeneous, while hyperechoic tumours were found in a few cases. Moreover, CDFI showed peripheral blood flow signals and no apparent blood flow into the tumours [6]. Different from previous reports, except for 3 cases, the majority of the tumours in the present study had well-defined margins and heterogeneous echo. A tumour with heterogeneous moderate echo was found in case no. 4. A cysticsolid mass with clear boundaries and a scattered anechoic and punctate strong echo was found in case no. 6. The tumour of case no. 7 showed ill-defined margins and was slightly heterogeneous hyperechoic with hypoechoic in the edge. CDFI showed a blood flow signal in the tumours of case nos. 4 and 6, while it showed blood flow around the tumour of case no. 1. We hypothesize that the reasons for this result were not only the bias of selection and the difference in diagnostic experience but also the mixed tumour composition especially the latter reason.

The CT imaging features of MTSCC were also included in the present study. Tumours were all solitary and either partially exophytic or parenchyma in the current case series with clear boundaries (capsule sign) on the CT scan, which is similar to previous reports [7, 8]. Compared with that of the normal renal parenchyma, a non-enhanced CT scan in the present study revealed isodense/slightly hypodense lesions in the majority of cases and 2 cases showed intralesional or central area calcification, mucous degeneration and cystic degeneration, while no intralesional or central area haemorrhage and fatty deposits were found, although these were shown in previous reports [7, 8]. The progressive and slow heterogeneous enhancement of MTSCC was described in previous studies [7, 8, 22, 25] but it could be homogenous when the tumour diameter is < 5 cm [7]. In the present case series, only 2 cases were found to be progressive with slow heterogeneous enhancement, including one small tumour with a 3.0 cm diameter, while the enhancement was quick with late washout in 3 cases, and 2 cases were found to be homogeneous, slow and progressive. We hypothesize that the progressive heterogeneous enhancement is due to the presence of variable amounts of extracellular mucin in the stroma of the tumour. Calcification, mucous degeneration and cystic degeneration were also found in case no. 6, for which the tumour was misdiagnosed as renal oncocytoma due to the progressive enhancement of mucous degeneration and cystic degeneration.

The enhancement pattern is the most valuable parameter in differentiating RCC subtypes [26–28]. Based on imaging findings, MTSCC was generally considered to be a hypovascular renal tumour. Maximum enhancement of MTSCC on CT image occurs in the nephrographic or excretory phase, which is different from clear cell RCC. Clear cell RCC is considered a hypervascular tumour commonly with heterogeneous enhancement, which is increased compared with that of the renal cortex. There is an overlap in the imaging characteristics of MTSCC and other hypovascular RCC subtypes (including chromophobe RCC and papillary RCC). Hence, it is still a challenge to distinguish MTSCC from other hypovascular RCCs using imaging techniques alone.

The majority of patients reported in the previous studies [6, 16, 25, 29] were metastasis-free, with only a few patients [7, 15, 29] presenting with pulmonary metastases, bone metastases and lymph node metastases, which indicates that MTSCC is an indolent renal cancer with a low mortality rate. In the present case series, none of the seven patients with MTSCC had metastases. Details are as follows: three patients were diagnosed with pT1aN0M0 (42.9%), two with pT1bN0M0 (28.5%), one with pT2bN0M0 (14.3%) and one with pT3bN0M0 stage with tumour thrombus in the inferior vena cava and left renal vein (14.3%), which is similar to the result of previous study [6]. However, presentation with locally advanced disease could be seen in a subset of the current patients. This warrants close follow-up even for localized tumours. The exact postoperative recurrence rate of MTSCC remains unclear. Based on a literature review, most cases had no recurrence after surgery [6–8, 20], only a few cases mentioned postoperative recurrence [30–33], and some cases were not available, died of MTSCC or other diseases during the follow-up [6, 7, 9, 29]. Therefore, the surgical recurrence rate cannot be accurately predicted due to the lack of accurate data in previous reports.

However, the present study has some limitations. First, the sample size is small, which is due to the low incidence rate of MTSCC. Second, no patient in this study undergo MRI examinations, although MRI images may provide more information in the diagnosis of MTSCC. Third, the surgical recurrence was not mentioned in the present study, although this may be due to the small sample size, the short follow-up time in some cases and the low malignancy of MTSCC. We speculate that the most likely reason is the low malignancy of MTSCC. In particular, some cases were followed up more than ten years in the present study without recurrence, which confirmed that MTSCC is a low malignant tumour. Therefore, we can also predict that the surgical recurrence rate of MTSCC is low and the effect of surgical treatment is very good.

In conclusion, MTSCC is not universally indolent and tends to occur in females of a broad age range. MTSCC is a hypovascular renal tumour, which is distinct from clear cell RCC. However, it is difficult to distinguish MTSCC from other hypovascular tumours because of the overlap in the imaging characteristics of MTSCC and other hypovascular RCC subtypes. More studies are needed to adequately describe the characteristics of MTSCC.

## Data Availability

The datasets used and/or analyzed during the current study are available from the corresponding author on reasonable request.
